# Network analysis for estimating standardization trends in genomics using MEDLINE

**DOI:** 10.1186/s12874-022-01740-4

**Published:** 2022-10-07

**Authors:** Eun Bit Bae, Sejin Nam, Sungin Lee, Sun-Ju Ahn

**Affiliations:** 1 Institute of Quantum Biophysics, Sungkyunkwan University, 16419 Seoul, Gyeonggi-do Republic of Korea; 2 Department of Psychiatry, Research Institute for Medical Bigdata Science, Korea University Anam Hospital, Seoul, Republic of Korea; 3 Department of Global Convergence, Sungkyunkwan UniversityR&D Center, ezCaretech Co., Ltd, 03063 Seoul, Jongno-gu, Republic of Korea

**Keywords:** Network analysis, Standard, Genomics, Genomic sequence, Keyword analysis, PubMed

## Abstract

**Background:**

Biotechnology in genomics, such as sequencing devices and gene quantification software, has proliferated and been applied to clinical settings. However, the lack of standards applicable to it poses practical problems in interoperability and reusability of the technology across various application domains. This study aims to visualize and identify the standard trends in clinical genomics and to suggest areas on which standardization efforts must focus.

**Methods:**

Of 16,538 articles retrieved from PubMed, published from 1975 to 2020, using search keywords “genomics and standard” and “clinical genomic sequence and standard”, terms were extracted from the abstracts and titles of 15,855 articles. Our analysis includes (1) network analysis of full phases (2) period analysis with five phases; (3) statistical analysis; (4) content analysis.

**Results:**

Our research trend showed an increasing trend from 2003, years marked by the completion of the human genome project (2003). The content analysis showed that keywords related to such concepts as gene types for analysis, and analysis techniques were increased in phase 3 when US-FDA first approved the next-generation sequencer. During 2017–2019, oncology-relevant terms were clustered and contributed to the increasing trend in phase 4 of the content analysis. In the statistical analysis, all the categories showed high regression values (R^2^ > 0.586) throughout the whole analysis period and phase-based statistical analysis showed significance only in the Genetics terminology category (*P* = .039^*^) at phase 4.

**Conclusions:**

Through comprehensive trend analysis from our study, we provided the trend shifts and high-demand items in standardization for clinical genetics.

**Supplementary Information:**

The online version contains supplementary material available at 10.1186/s12874-022-01740-4.

## Introduction

The dawn of the 20th century saw the rise of medical genetics research on humans due to the discovery of Mendelian inheritance disorders [1, 2]. Remarkable progress in medical genetics has been made in the latter part of the 20th century, notably in cancer genetics [3]. Especially, research on disease diagnosis using genomic sequencing technologies has gained momentum, thanks to the wide availability of next-generation sequencing (NGS) methods. To use this advanced genetic analysis technology in medical institutions or clinical settings, it is essential to develop a standard procedure that could be commonly used. Various standard guidelines are being developed by industry and international standards development organizations for clinical examination and diagnosis of diseases, such as cancer, leukemia, and tuberculosis [4–7]. These standards, from such organizations as the American College of Medical Genetics (ACMG), Association of Molecular Pathology, and Microarray Quality Control Consortium [4, 7–9], have enabled the active use of various sequencing technologies and methods in the clinic [7].

However, extant standards and their coverage could not be claimed to be sufficient to meet the standardization demand from the market, notably evident in the clinical applications of NGS to disease diagnosis [4–7]. For the use of newly developed genetic technology to thrive, it is of significant import for standard research to be able to scan the clinical environment of genomics and the recent status/ trend of analysis technology and gather necessary technical resources for standardization, to refine priorities for genomics standardization.

One way to help scan the genomics environment is to apply network analysis on the artifacts of research articles to reveal environmental changes that can be used as guidance for standardization. To explore specific research trends, network analysis using bibliometric data has been widely used and applied to various research domains, for example, genomics [10], public health [11, 12], and medicine [13]. Network analysis in this study is used to divulge research trend changes. The identification of such trend changes can enable the research of standards development to construct a strategy to meet the standardization demand from genetic research and clinical practice. In detail, this study uses network analysis (1) to suggest recent genomics trends and narrowed range of topics to keywords showing strong relation in standardization, (2) to examine temporal trends and related critical development which drives changes in trend. Through this study, we intend to derive all development that acts as major factors and indicators to which standards development should be considered.

## Methods

### Study flow

The overall study procedure is shown in [Supplementary file, Figure S1] and summarized as follows: (1) search articles with two Medical Subject Heading (MeSH) terms (“genomics and standard” and “clinical genomic sequence and standard”) in PubMed; (2) export PMID numbers; (3) extract keywords from the abstracts and titles of the articles; (4) keyword preparation; (5) development of the network analysis with the keyword frequency matrix; (6) development of the period analysis with the keyword frequency matrix; and (7) categorization of keywords for statistical analysis.

### Data source

The MEDLINE database is provided by the US National Library of Medicine and contains various types of scientific literature in biomedical and life science fields [14]. We have used PubMed to freely access to MEDLINE database, and it provides links to the abstracts. To explore research trends of standardization in genomics, we searched two MeSH terms, “genomics and standard” and “clinical genomic sequence and standard”, published between 1975 and September 2020. The search returned 16,550 articles that contained various types of research papers, such as reviews, original articles, and perspectives. Of the articles, 10,000 articles were indexed with the search term “Genomics and standard”, and 6,550 articles with “Clinical genomic sequence and standard”. Of the 16,550 articles, we used 15,855 articles whose abstracts and titles were accessible and written in English.

### Keyword preparation

The data preparation was summarized in [Supplementary file, Figure S1]. A total of 36,275 frequency of 5,639 keywords was extracted from 15,855 articles. The keywords were extracted using the TextRank algorithm [15] using Corpus 16,000 from the abstracts and titles of the articles. TextRank algorithm is commonly used to extract single terms from literature, so we used TextRank to extract semantic keywords. By four experts, the keywords were manually screened and reviewed following a set of exclusion criteria referring to previous studies. The exclusion criteria are 1) non-technical terms with three conditions: (a) everyday term which is used in daily life, such as “she”, and “others”. (b) terms that are not related to or specialized science and technical knowledge, such as “abc”, “scientist”, “concept” and “consensus”. (c) adjectives and adverbs, such as “happy”, “firstly”, “lastly”, and “furthermore”; 2) temporal terms such as months, weekdays, as well as other temporal terms that do not provide precise a point of time and period, such as “April” without year (instead of “April 2004”) or “Monday” without year and month; and 3) compound nouns with two conditions: (a) frequencies of a compound noun of whose constituent terms have been already counted individually, such as “genomics proteomics”, and “protein gene” AND (b) the compound noun does not constitute a meaningful term, such as “furthermore genes” and “statistically disease”. After the manual cleansing, 1,024 keywords were left.

For the synonyms with different spells and the synonyms expressed with different capital or small character, we merged these terms into one abbreviation of capital instead of a spell-out term. All the plural terms were corrected and merged into a singular form.

Because many duplicated compound nouns, such as “HBV HBC” “proteomics proteomics” and “CpG CpG”, and meaningless compound nouns with more than three words, such as “genorm bestkeeper normfinder” and “genetics genomics acmg”, were automatically generated under 12 frequencies, we set further exclusion criteria for keywords less than 12 frequencies. As we removed keywords following this exclusion criteria, most of the unuseful compound nouns were deleted and it resulted in 330 keywords with a total frequency of N = 16,213.

### Network analysis

The overall network analysis was performed following previous studies [16, 17]. In network analysis of research articles, a higher frequency of keywords indicates a higher number of relevant research in a particular year. For network analysis, weighted Jaccard similarity value obtained between two keywords was commonly used to evaluate the closeness between the keywords. A network consists of lots of nodes and edges. A node represents a keyword, and an edge represents relatedness between two keywords.

The weighted Jaccard similarity provides edge weight 0 to 1. For example, if the edge weight is 1, two keywords were always used in the same sentence. In this study, we calculated edge, the relatedness between two keywords, by weighted Jaccard similarity using frequencies of the keywords [16, 17]. For network analysis, we used keywords frequency data in the full phase. The weight of a node in the network was determined by the PageRank algorithm [18], and a community detection algorithm [19] were used to cluster keywords. When PageRank calculates node sizes, it considers edge weights. In this study, the PageRank, and the community detection algorithm based on the modularity of optimization were conducted via Gephi 0.8.2. The node size was displayed by the PageRank score, and the color of an edge was presented by the modularity value. According to the derived values, the network model of the relationships between keywords was visualized via Gephi.

The similarity between keywords and between publication year.

The relatedness between keywords is represented by the similarity obtained via the weighted Jaccard similarity equation shown below.

$$J\left(S, T\right)=\frac{{\sum }_{K}\text{min}\left(S_\text{K},T_\text{K}\right)}{{\sum }_{K}\text{m}\text{a}\text{x}(\text{S}_\text{K},\text{T}_\text{K})}$$

First, a two-dimensional annual frequency matrix (Supplementary file 1, Figure S1) was generated with a frequency of each term by publication years - a matrix of 330 (the number of keywords) x 46 (the number of publication years, from 1975 to 2020). In the following equation, for the network analysis, S and T represent two keywords, and $\text{K}$represents the ordinal number of keywords S and T. Based on the matrix, we calculated the similarity value between the two keywords using frequency data in a row. For example, when we calculate similarity between keywords “AAV (S)” and “Abi (T)”, the frequency data for the keywords are: S = {0, …, 1, 0} and T = {1, …, 1, 0}. Using these input data, we obtained the similarity value of *J* (S, T) = (0 + … + 1 + 0)/(1 + … + 1 + 0). For the period analysis, we used frequency data in a column of each publication year to calculate the similarity between publication years. For example, the similarity between 2019:2020 is calculated with the frequency of 2019 (S) and 2020 (T): S = {1, 1, 0, 1, 8, 2, 4, 0, …} and T = {0, 0, 1, 1, 2, 0, 0, 0, …}. Thus, the similarity value between 2019:2020 is *J* (S, T) = (1 + 1 + 0 + 1 + 8 + 2 + 4 + 0 + …)/(0 + 0 + 1 + 1 + 2 + 0 + 0 + 0 + …). The maximum similarity value is 1.0, and as the similarity is increasing, two keywords in the network analysis or two publication years in the period analysis present a high match.

### Period analysis

To observe when the research trend changed, a similarity analysis was performed between years. Through period analysis, we identified the change point when the similarity graph was steeply curved. This will aid in exploring the social events that affect research trends. We calculated the differences between the year of similarities to identify the local minimum and the local maximum points. Before and after of the relatively larger difference value [red color in Supplementary file 2], the local minimum and maximum points were identified [Supplementary file 2, green colored].

To be more precise about the local minimum and maximum points, we analyzed three types of similarity analysis for the period analysis:

1) The similarity between two publication years (e.g., years 2000 and 2001 presented as 2000:2001).

2) The similarity between two similarity values with 1-year of interval (e.g., similarity between similarity values of 2000:2001 and 2001:2002 presented as 2000:2001:2001: 2002).

3) The similarity between two similarity values with 2-year of interval (e.g., similarity between similarity values of 2000:2002 and 2001:2003).

Please note that phase 0 (1975–1999) was not included in the analysis, due to the low-frequency values (frequency of 10 to 72).

We submit that a local minimum and maximum point in similarity provides an indicator that there has been a significant development or event that deserves the attention of standards development communities.

### Content analysis

Through content analysis, we reviewed terms following our previous research [16, 17], and additionally, in this study, we classified keywords into a related research area. First, the 330 keywords were classified into academic categories, and further, the same 330 keywords were classified into other subcategories [Supplementary file 2, Content analysis sheet]: 1) The keywords were sorted into six academic categories: Biology, General, Genetics, Medicine, Proteomics, and Statistics. For example, “Escherichia”, “animal”, and “Arabidopsis” were sorted into the Biology, “Illumina”, “allele”, and “rRNA” were in the Genetics, “precision”, “therapy”, and “diagnosis” were in the Medicine, “peptide” “omics”, and “QconCAT” were in the Proteomics, “Bayesian”, “algorithm”, and “Gaussian” were in the Statistics. The keywords in the General category can be used in other academic fields. For example, “database” “knowledge” and “measurement” could be used in any other field in Biology, Genetics, and Medicine. So in this case, keywords were classified into the General category.

2) Further, those 330 keywords were divided into the 12 science subcategories: Biologicals/Metabolics, Clinical, Company/Consortium, Database/ Software, Disease, Gene, Genetics term, Methods, Organism, Pathogen, Proteomics, and Statistics.

All the keywords category lists were in [Supplementary file 2, Content analysis sheet].

### Statistical analysis

To evaluate statistically linear trends, the generalized linear model has been commonly used in review and research articles [20, 21]. In our study, a linear regression analysis was performed with keyword frequencies and publication years for each category to examine the relationship between phases. The sum of the publication year within a phase was calculated in the five phases, including phase 0 to derive phase-frequency data. The academy categories and subcategories were represented as fixed factors. And the five phase-frequency lists were used as the dependent variables. Using these variables, we performed a univariate generalized linear model (GLM) to statistically estimate the research trends of each phase. For the GLM, we conducted a parameter estimation in each of the 6 academy categories and 12 subcategories within each phase. SPSS Statistics ver.26, IBM was used for the statistical analysis.

## Results

### The network analysis

**Fig. 1:**
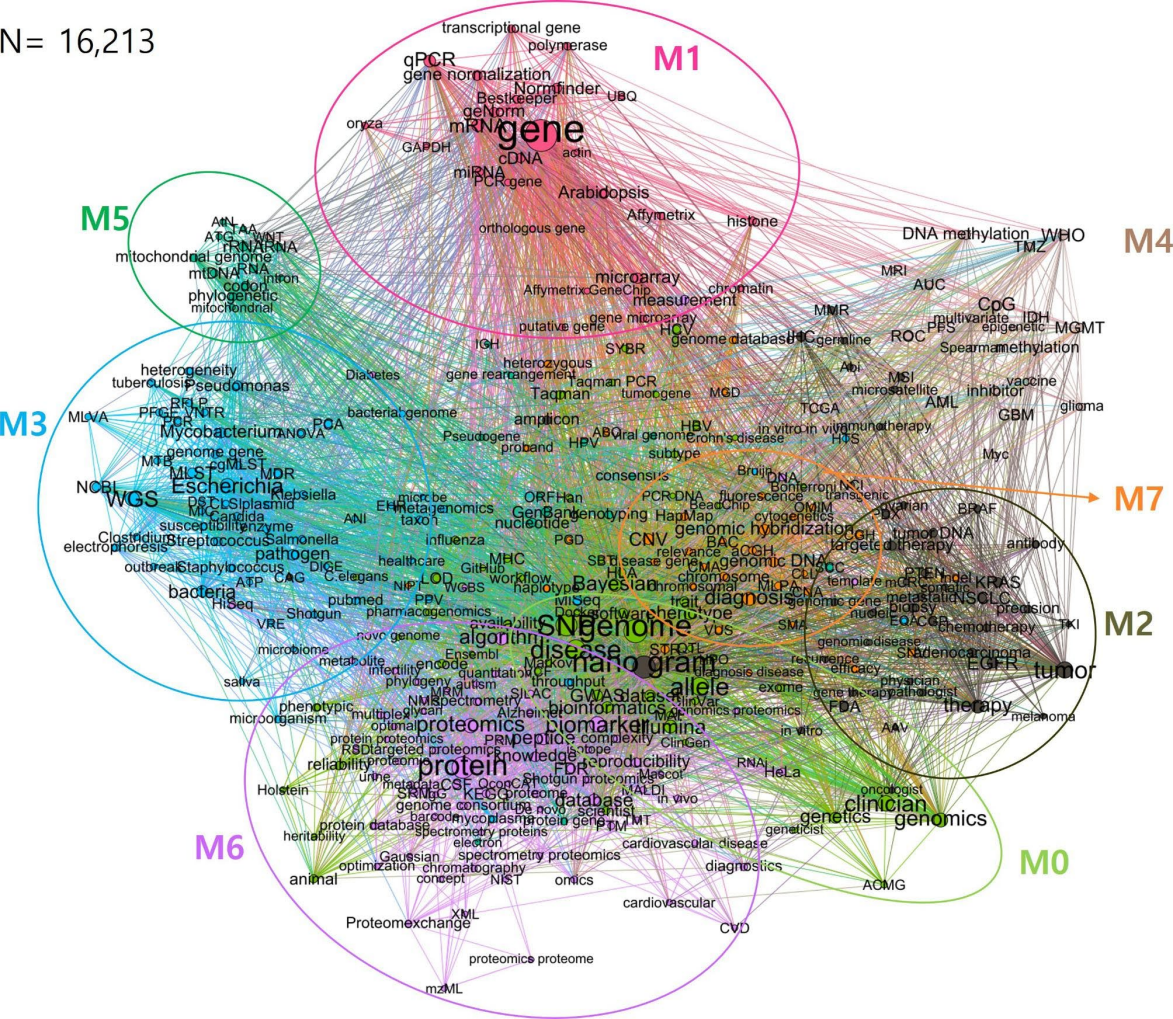
Network connectivity between keywords for the total period (1975–2020). In the network, the total frequency of 338 keywords is 16,213. The color of an edge represents the same similarity value and represents the cluster. Each keyword has one node and a keyword, and it may have many edges to and from other keywords. A node size was determined by a PageRank score. The edge is displayed over 0.5 threshold of modularity

The network is displayed in Fig. 1 with keywords derived from studies published from 1975 to 2020, using eight colors following a modularity of 0 to 7. According to the modularity value, full-phase keywords were clustered in different colors (Fig. 1; Table 1).

**Table 1:** The top keyword lists and the PageRank scores by the modularity number of the cluster

**Modularity 0**		**M1**		**M2**		**M3**	
**Keyword**	**PageRank**	**Keyword**	**PageRank**	**Keyword**	**PageRank**	**Keyword**	**PageRank**
genome	0.0167	Gene	0.0244	nano gram	0.0159	WGS	0.0087
SNP	0.0148	mRNA	0.0067	tumor	0.0105	Escherichia	0.0084
disease	0.0128	qPCR	0.0066	therapy	0.0076	bacteria	0.0061
allele	0.0119	microarray	0.0052	EGFR	0.0056	pathogen	0.0053
clinician	0.0097	Arabidopsis	0.0048	IHC	0.0043	Mycobacterium	0.0051
genomics	0.0084	geNorm	0.0048	KRAS	0.0041	MLST	0.0047
Illumina	0.0075	gene normalization	0.0047	NSCLC	0.0041	NCBI	0.0039
Bayesian	0.0072	NormFinder	0.0045	targeted therapy	0.0040	MiSeq	0.0038
genetics	0.0058	cDNA	0.0042	amplicon	0.0037	Pseudomonas	0.0038
bioinformatics	0.0058	miRNA	0.0035	tumor DNA	0.0037	Streptococcus	0.0037
**M4**		**M5**		**M6**		**M7**	
**Keyword**	**PageRank**	**Keyword**	**PageRank**	**Keyword**	**PageRank**	**Keyword**	**PageRank**
CpG	0.0053	rRNA	0.0042	protein	0.0152	diagnosis	0.0077
WHO	0.0051	nucleotide	0.0038	biomarker	0.0095	CNV	0.0056
DNA methylation	0.0040	GenBank	0.0037	proteomics	0.0087	genomic hybridization	0.0054
methylation	0.0037	codon	0.0036	Algorithm	0.0075	genomic DNA	0.0047
MGMT	0.0036	genotyping	0.0033	Peptide	0.0063	STR	0.0032
inhibitor	0.0036	mitochondrial genome	0.0031	Database	0.0060	chromosome	0.0031
AML	0.0036	mtDNA	0.0031	knowledge	0.0047	BAC	0.0030
ROC	0.0034	phylogenetic	0.0031	reproducibility	0.0046	aCGH	0.0029
TMZ	0.0032	tRNA	0.0031	FDR	0.0040	MLPA	0.0028
IDH	0.0031	RNA	0.0029	measurement	0.0040	Haplotype	0.0028

In the modularity 0 (M0), terms related to genetic materials (e.g., “genome”, “SNP”, and “allele”), clinical related terminology (e.g., “disease”, “clinician”, “Illumina”), and bioinformatics technology (e.g., “Bayesian”, “bioinformatics”) are clustered. M0 is implied that bioinformatics and its technology are applied in clinics for the detection and examination of various types of genes. In M1, object of genetic analysis and its techniques are clustered; The analysis subjects including genetic materials were “gene”, “mRNA”, “Arabidopsis”, “cDNA” and “miRNA”.) and gene analysis terms were “qPCR”, “microarray”, “GeNorm”, “gene normalization”, and “NormFinder”). In M2, top ranked keywords are used most in clinics to diagnose and treat tumor diseases; The histo-technical terms to detect tumor genes from tumor cell and tissue, such as “nano gram”, “EGFR”, “IHC”, and “KRAS”, tumor related keywords (“tumor”, “NSCLC” and “tumor DNA”), and treatment keywords (“therapy”, and “targeted therapy”) are grouped together. In M3, strong relations are shown among gene database (“MLST” and “NCBI”), genetic analysis (“WGS” and “MiSeq”) and pathogens (“Escherichia”, “bacteria”, “pathogen”, “Mycobacterium”, “Psudomonas”, and “Streptococcus”). In M4, The DNA methylation-related disease and its specific genetic analysis terminology are clustered; DNA methylation-related term (“CpG”, “DNA methylation”, “methylation”, “MGMT”), DNA methylation disease (“AML”), and the specific terms (“TMZ”, “IDH”) regarding glioblastoma which is one of the DNA methylation diseases. In M5, gene-related terms (“rRNA”, “nucleotide”, “GenBank”, “codon”, “genotyping” “RNA”) are shown in the cluster including genes for phylogenetics (“mitochondrial genome”, “mtDNA”, “phylogenetic”, and “tRNA”). In M6, proteomics (“protein”, “proteomics”, and “peptide”) and its analytical terms (“biomarker”, “algorithm”, “database”, “knowledge”, “reproducibility”, “FDR”, “measurement”) are clustered. The keywords in M7 are considered that these are related to the subjects of genetic analysis in clinical laboratory; clinical laboratory techniques (“diagnosis”, “genomic hybridization” “aCGH”, and “MLPA”), and the subjects of analysis (“CNV”, “genomic DNA”, “STR”, “Chromosome”, “BAC”, “aCGH”, “MLPA”, and “haplotype”). From the network analysis, it was possible to explore keywords related standards and a field of genetics research where standardization is mentioned.

### Period analysis based on publication years

**Fig. 2:**
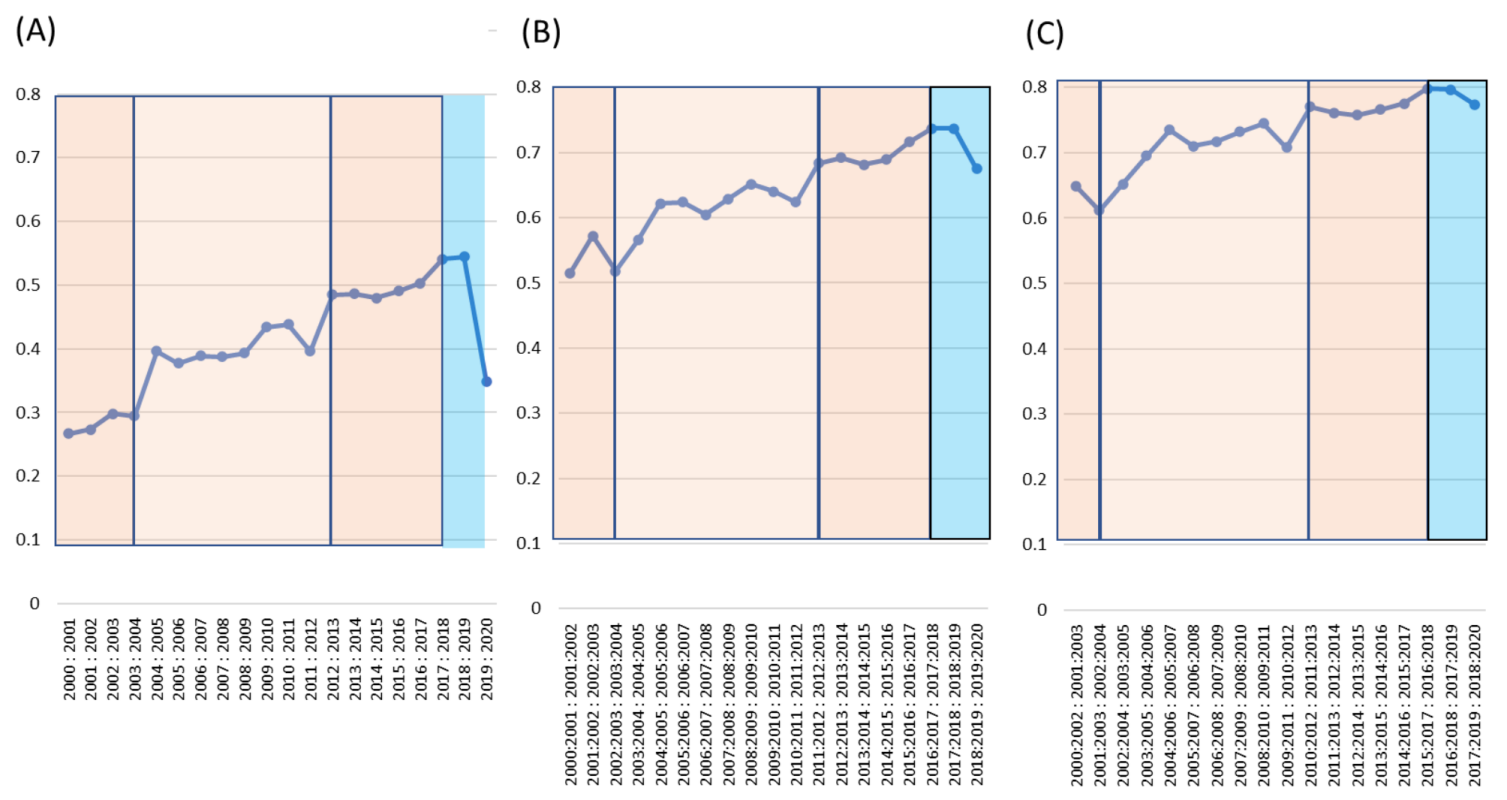
Period analysis. **(A)** The similarity between 1-year; **(B)** similarity between a 1-year interval of similarities; **(C)** similarity between a 2-year interval of similarities.

For period analysis, we selected three local minimum / maximum points using a large difference between the similarities of publication year [Supplementary file 2] to define the patterns of the keyword appearance. Based on the local minimum and maximum points, four phases were defined for the different similarity patterns shown in the keyword research. For example, the local minimum points (Similarity = 0.294) were identified in 2003:2004 in Fig. 2 A, S = 0.518 in 2002:2003:2003:2004 in Fig. 2B, and S = 0.612 in 2001:2003:2002:2004 in Fig. 2 C. So, phase 1 was set from 2000 to 2003 based on the local minimum points near the large difference (-0.102, in Fig. 2 A).

Following the procedure, in Fig. 2 A, the local minimum / maximum points emerged in 2003:2004, 2012:2013 (S = 0.485), and 2017:2018 (S = 0.541) where the trend has started to plateau (Table 2). In the same way, the phase criteria of Fig. 2B were defined as 2002:2003:2003:2004 (S = 0.518); 2011:2012:2012:2013 (S = 0.684); and 2016:2017:2017: 2018 (S = 0.736), and Fig. 2 C were 2001:2003:2002:2004 (S = 0.612); 2010:2012:2011: 2013 (S = 0.770); and 2015:2017:2016:2018 (S = 0.798). The similarity scores for each period analysis are shown in Table 2. From above the periodic analysis, we identified the main three points, where the critical issues regarding standardization in genomics occurred.

**Table 2:** Similarity results based on different year ranges

Similarity (1 year)		Similarity (1-year interval)			Similarity (2-year interval)		
Year	Similarity	Year	Similarity	Year		Similarity	
2000 : 2001	0.267	2000:2001 : 2001:2002	0.515	2000:2002 : 2001:2003		0.649	
2001 : 2002	0.274	2001:2002 : 2002:2003	0.572	2001:2003 : 2002:2004		0.612	
2002 : 2003	0.298	2002:2003 : 2003:2004	0.518	2002:2004 : 2003:2005		0.652	
2003 : 2004	0.294	2003:2004 : 2004:2005	0.566	2003:2005 : 2004:2006		0.695	
2004 : 2005	0.396	2004:2005 : 2005:2006	0.622	2004:2006 : 2005:2007		0.735	
2005 : 2006	0.377	2005:2006 : 2006:2007	0.624	2005:2007 : 2006:2008		0.710	
2006 : 2007	0.389	2006:2007 : 2007:2008	0.605	2006:2008 : 2007:2009		0.717	
2007 : 2008	0.387	2007:2008 : 2008:2009	0.628	2007:2009 : 2008:2010		0.732	
2008 : 2009	0.393	2008:2009 : 2009:2010	0.651	2008:2010 : 2009:2011		0.745	
2009 : 2010	0.434	2009:2010 : 2010:2011	0.640	2009:2011 : 2010:2012		0.708	
2010 : 2011	0.439	2010:2011 : 2011:2012	0.624	2010:2012 : 2011:2013		0.770	
2011 : 2012	0.397	2011:2012 : 2012:2013	0.684	2011:2013 : 2012:2014		0.761	
2012 : 2013	0.485	2012:2013 : 2013:2014	0.692	2012:2014 : 2013:2015		0.757	
2013 : 2014	0.486	2013:2014 : 2014:2015	0.682	2013:2015 : 2014:2016		0.766	
2014 : 2015	0.480	2014:2015 : 2015:2016	0.689	2014:2016 : 2015:2017		0.775	
2015 : 2016	0.491	2015:2016 : 2016:2017	0.716	2015:2017 : 2016:2018		0.798	
2016 : 2017	0.503	2016:2017 : 2017:2018	0.736	2016:2018 : 2017:2019		0.796	
2017 : 2018	0.541	2017:2018 : 2018:2019	0.737	2017:2019 : 2018:2020		0.774	
2018 : 2019	0.544	2018:2019 : 2019:2020	0.675				
2019–2020	0.349						

### Content analysis

The combined frequencies of keywords belonging to each category of the academic categories and subcategories are computed. Each keyword belongs to only one category.

Genetics in academic category has the highest frequency (n = 8,777, 54.1%) in the academic categories, followed by. Medicine (n = 2,856, 17.6%), Proteomics (n = 2,257, 13.9%), General (n = 992, 6.1%), Biology (n = 707, 4.3%), and Statistics (n = 624, 3.81%).

Gene in subcategories has the highest frequency (n = 3276, 20.2%), followed by Genetics terminology (n = 3019, 18.6%), Methods (n = 1725, 10.6%), Database/Software (n = 1393, 8.59%), Disease (n = 1204, 7.42%), Clinical (n = 1103, 6.8%), Proteomics (n = 1034, 6.37%), Pathogen (n = 1006, 6.2%), Statistics (n = 720, 4.44%), Biologicals (n = 707, 4.36%), Company/Consortium (n = 536, 3.3%), and Organism (n = 490, 3.02%).

We examined the trend of each term from phase 0 to phase 4 in subcategories as follows:

In [Supplementary file 1, Figure S2], “Escherichia” showed the highest frequency in phase 2, and “Mycobacterium” in phase 4. In Statistics, “Bayesian” and “algorithm” were of the highest frequency in phase 2, while the frequency of the latter steadily decreased until phase 4. The frequency of “Bayesian” increased from phase 3 to 4.

In the Company/Consortium graph, “Illumina,” “Taqman” were of the highest frequency at phase 4, and “Illumina” and “ACMG” showed an increasing trend during the whole period. In Database, the term “bioinformatics” showed the highest frequency at phase 4. In Gene, the terms “gene”, “genome”, “allele”, “codon”, “cDNA”, “chromosome”, “DNA”, and “mtDNA” exhibited the highest frequencies at phase 2 and started to decrease in frequency from phase 3 to phase 4.

Terms denoting relatively smaller gene fragments, such as “RNA”, “miRNA”, “rRNA”, “exome”, “tRNA”, showed an increasing trend from phase 3 to 4. In Software, terms referring to gene quantification software, “NormFinder”, “geNorm”, and “BestKeeper”, were highest in frequency at phase 3 and “ClinGen” showed an increasing trend from phase 3 to 4. In Methods, “WGS”, “GWAS”, and “MiSeq” exhibited an increasing trend from phase 2 and peaked in frequency at phase 4.

On the other hand, “microarray,” “genomic hybridization,” and “gene microarray” showed the highest frequency in phase 2, and “qPCR” peaked in frequency in phase 3. In Clinical, “Clinician”, “therapy”, “diagnosis”, “precision”, “targeted therapy”, and “biopsy” all showed an increasing trend until phase 4; and in Disease, the term “disease” and oncology-related terms, such as “tumor”, “NSCLC”, “AML”, “GBM”, “tumor DNA”, and “adenocarcinoma” showed an increasing trend throughout the phases.

### Statistical analysis

#### The linear regression over the period

To evaluate linear trends, linear regression was conducted with keyword frequencies for publication years from 1975 to 2020. Although 2020 showed a decreasing trend in the academic categories and subcategories, all the categories showed high regression values (from 0.586 (Company/Consortium) to 0.764 (Biology)) as shown in Table 3; Fig. 2. All the categories showed an increasing linear correlation between keyword frequencies and publication years.

**Fig. 3:**
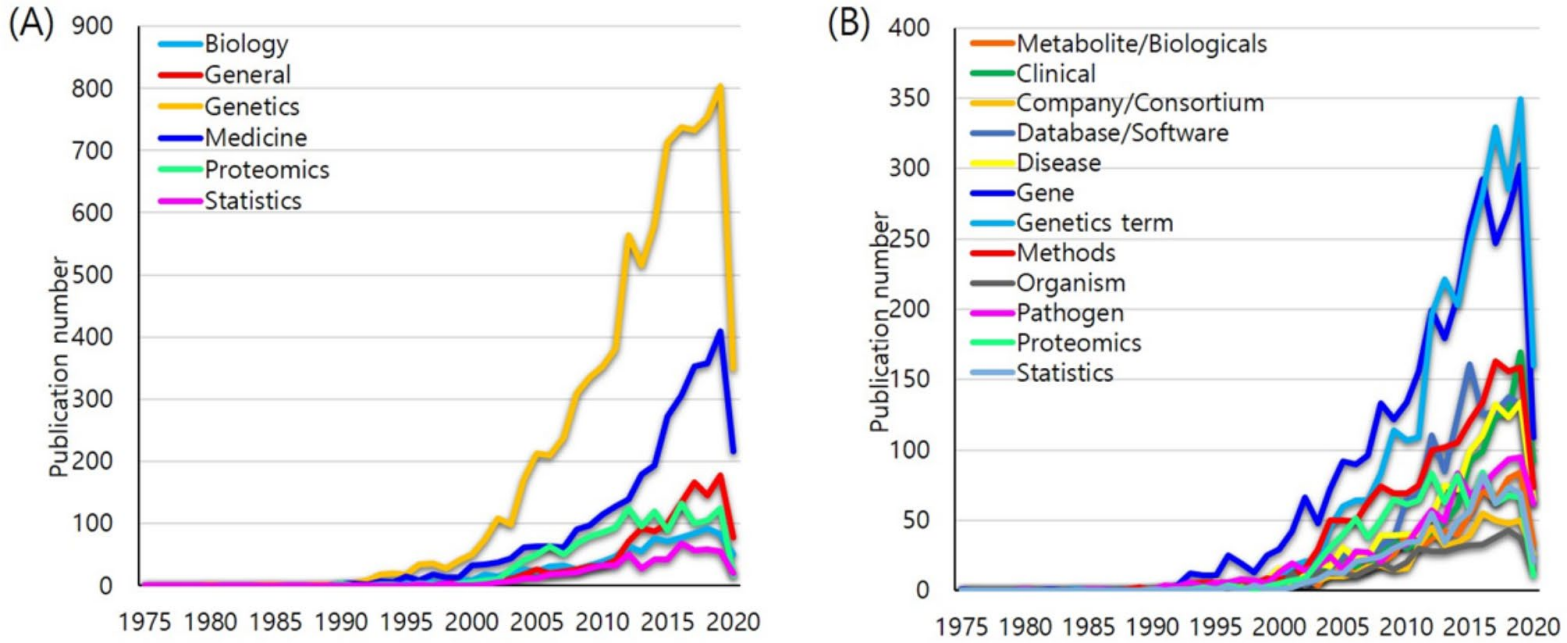
Keyword frequency trend results. **(A)** publication frequencies’ trend of the academic categories; **(B)** publication frequencies’ trend of subcategories

**Table 3:** Linear regression based on keyword frequency in the academic categories and subcategories

	Name of the category	R^2^
Academic Category	Biology	0.764
	General	0.587
	Genetics	0.717
	Medicine	0.653
	Proteomics	0.673
	Statistics	0.666
Subcategory	Clinical	0.657
	Company/Consortium	0.586
	Database/software	0.684
	Disease	0.625
	Gene	0.664
	Genetics terminology	0.740
	Metabolite/Biologicals	0.652
	Methods	0.736
	Organism	0.741
	Pathogen	0.737
	Proteomics	0.678
	Statistics	0.648

#### The generalized linear model within a phase

Because the linear regression analysis without phase demonstrated a high correlation (R^2^ > 0.585) in all categories, we conducted linear regression within a phase in each category. To analyze phase-based linear analysis for each category, we performed GLM evaluation based on phases (Fig. 3; Table 4). There was no significant linear correlation found in the academic categories (Supplementary file 1, Table S1) while significant linear correlations were observed in several subcategories (Table 4): Gene (*P* = .003) and Pathogen (*P* = .030) showed a significant in phase 0, and Gene (*P* = .004) and Proteomics (*P* = .044) showed a significant phase 1. In phase 2, only Proteomics (*P* = .001) was significant, in phase 3, Proteomics (*P* = .045) and Software (*P* = .004) were significant, and in phase 4, only Genetics terminology was significantly fitted with the linear model (*P* = .039).

**Fig. 4:**
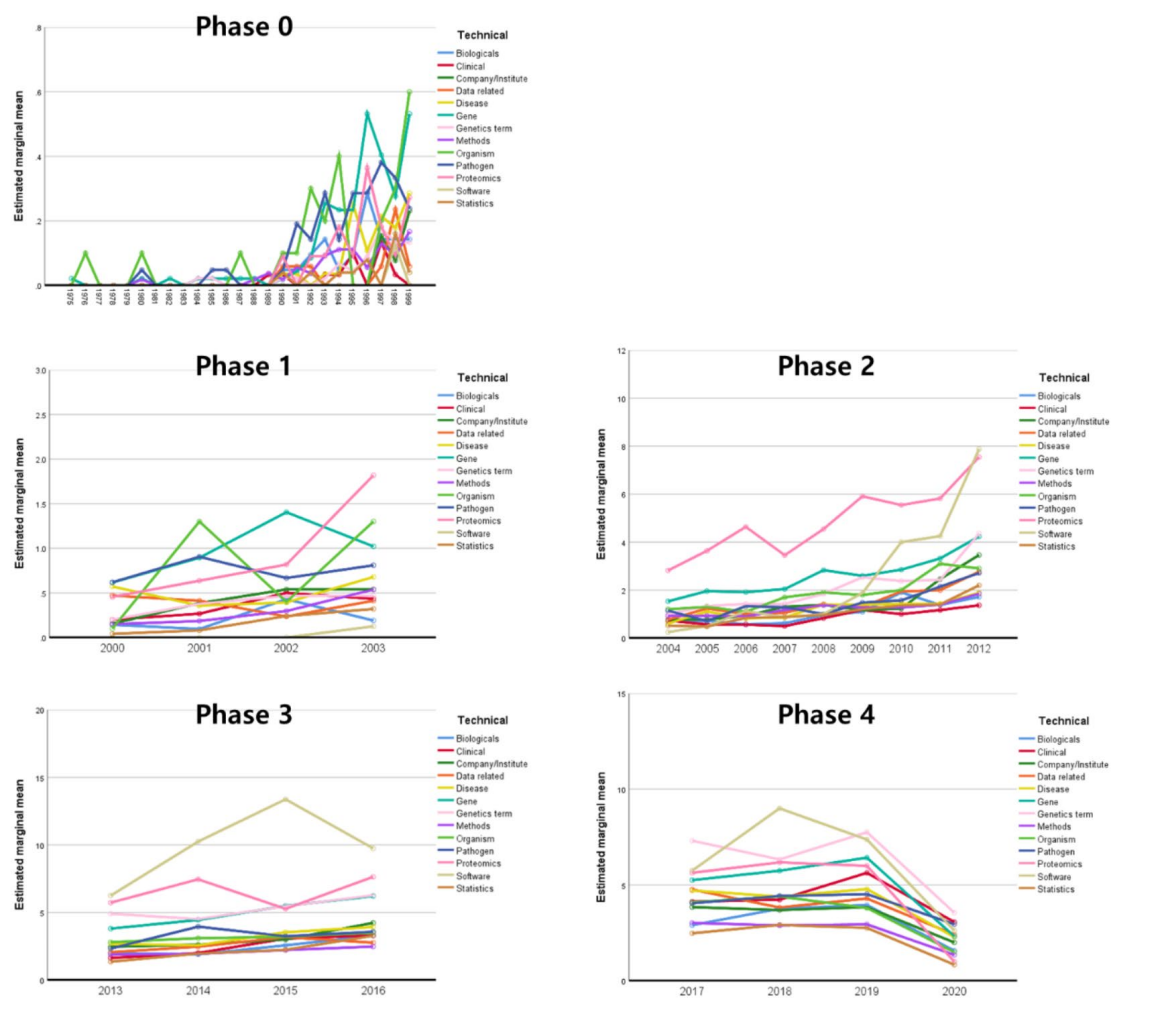
The generalized linear model results represented each phase from phase 0 to phase 4

**Table 4:** Generalized linear model results of subcategories from phase 0 to phase 4

Phase	Category		B	SE	t	Sig.	95% Confidence Interval		
							Lower	Upper	
Phase 0	Biologicals		0.647	0.911	0.710	0.478	-1.146	2.439	
	Clinical		0.004	0.875	0.005	0.996	-1.717	1.726	
	Company/Institute		0.022	1.078	0.020	0.984	-2.100	2.143	
	Data related		0.089	0.991	0.090	0.928	-1.861	2.040	
	Disease		0.739	0.868	0.851	0.395	-0.969	2.446	
	Gene		2.347	0.781	3.007	0.003^**^	0.812	3.883	
	Genetics terminology		0.299	0.783	0.382	0.703	-1.242	1.841	
	Methods		0.504	0.763	0.661	0.509	-0.996	2.005	
	Organism		2.338	1.226	1.907	0.057	-0.074	4.749	
	Pathogen		2.036	0.933	2.182	0.030^*^	0.200	3.873	
	Proteomics		1.160	1.180	0.983	0.326	-1.161	3.481	
	Software		-0.315	1.281	-0.246	0.806	-2.835	2.205	
Phase 1	Biologicals		0.146	1.305	0.112	0.911	-2.422	2.714	
	Clinical		0.876	1.254	0.698	0.485	-1.591	3.342	
	Company/Institute		0.935	1.544	0.606	0.545	-2.103	3.974	
	Data related		0.849	1.420	0.598	0.550	-1.944	3.643	
	Disease		1.320	1.243	1.062	0.289	-1.125	3.765	
	Gene		3.256	1.118	2.912	0.004^**^	1.056	5.456	
	Genetics terminology		0.798	1.122	0.711	0.477	-1.410	3.006	
	Methods		0.487	1.093	0.445	0.656	-1.663	2.636	
	Organism		2.653	1.756	1.511	0.132	-0.801	6.108	
	Pathogen		2.320	1.337	1.735	0.084	-0.311	4.951	
	Proteomics		3.420	1.690	2.024	0.044^*^	0.095	6.745	
	Software		-0.555	1.835	-0.303	0.762	-4.165	3.055	
Phase 2	Biologicals		-0.318	8.863	-0.036	0.971	-17.757	17.120	
	Clinical		-1.951	8.514	-0.229	0.819	-18.704	14.801	
	Company/Institute		3.622	10.490	0.345	0.730	-17.017	24.260	
	Data related		3.631	9.644	0.376	0.707	-15.343	22.605	
	Disease		1.089	8.441	0.129	0.897	-15.519	17.697	
	Gene		13.437	7.594	1.769	0.078	-1.504	28.377	
	Genetics terminology		8.486	7.622	1.113	0.266	-6.511	23.483	
	Methods		1.530	7.421	0.206	0.837	-13.070	16.131	
	Organism		8.160	11.925	0.684	0.494	-15.303	31.623	
	Pathogen		3.541	9.080	0.390	0.697	-14.325	21.407	
	Proteomics		38.460	11.478	3.351	0.001^**^	15.877	61.043	
	Software		11.910	12.461	0.956	0.340	-12.607	36.427	
Phase 3	Biologicals	0.421		7.619	0.055	0.956	-14.570	15.412	
	Clinical	1.493		7.319	0.204	0.838	-12.908	15.894	
	Company/Institute	3.468		9.017	0.385	0.701	-14.274	21.209	
	Data related	1.631		8.290	0.197	0.844	-14.680	17.941	
	Disease	3.874		7.256	0.534	0.594	-10.403	18.151	
	Gene	11.117		6.528	1.703	0.090	-1.726	23.961	
	Genetics terminology	11.921		6.552	1.819	0.070	-0.971	24.813	
	Methods	-0.081		6.379	-0.013	0.990	-12.632	12.471	
	Organism	4.938		10.251	0.482	0.630	-15.232	25.107	
	Pathogen	4.255		7.806	0.545	0.586	-11.103	19.614	
	Proteomics	19.860		9.867	2.013	0.045^*^	0.446	39.274	
	Software	30.785		10.712	2.874	0.004^**^	9.709	51.861	
Phase 4	Biologicals	2.348		8.725	0.269	0.788	-14.818	19.514	
	Clinical	9.000		8.381	1.074	0.284	-7.490	25.490	
	Company/Institute	4.385		10.326	0.425	0.671	-15.931	24.700	
	Data related	6.294		9.493	0.663	0.508	-12.383	24.971	
	Disease	7.179		8.309	0.864	0.388	-9.170	23.527	
	Gene	10.745		7.475	1.437	0.152	-3.963	25.452	
	Genetics term	15.587		7.503	2.077	0.039^*^	0.824	30.350	
	Methods	1.537		7.305	0.210	0.833	-12.835	15.909	
	Organism	5.778		11.738	0.492	0.623	-17.318	28.873	
	Pathogen	6.952		8.938	0.778	0.437	-10.634	24.539	
	Proteomics	11.700		11.299	1.036	0.301	-10.530	33.930	
	Software	15.750		12.256	1.284	0.200	-8.384	39.884	

## Discussion

In this study, we have investigated the trends in clinical genetics from 1975 to 2020. Through the network analysis, we have obtained clusters with a strong relationship between terminology from M0 to M7 as follows, respectively: M0) clinical use of bioinformatics and analysis technology; M1) gene analysis objects, methods, and software; M2) oncology regarding diagnosis, treatment, and tumor disease; M3) gene database and analysis tools regarding pathogens; M4) The DNA methylation-related disease and gene analysis; M5) gene-related terms including phylogenetics; M6) proteomics and its analytical terms; M7) gene analysis objects in clinical laboratory. As the clinical application of cutting-edge technology increases, research items with high requirements for standardization are being revealed, and the scope seems to be narrowing down to gene analysis, genetic materials, living organisms (i.e., biological objects), bioinformatics, and proteomics. Interestingly, diseases in which standardization is often mentioned or is showing high demands for standards are prominent in clinical practice have been discovered, such as oncological diseases such as tumors and cancer, and DNA methylation diseases such as acute myeloid leukemia (AML) and glioblastoma.

Through period analysis, it was possible to know at which point the standard trend in the field of clinical genetics changed, and through content analysis, it was possible to find out which keywords increased at the point revealed through period analysis.

For instance, in April 2003, the Human Genome Project, the world’s largest collaborative biological project from 1990, was completed [22], ramifications of which seemed to have been reflected in the trend shift at phase 2. In a comprehensive review of the content analysis and network analysis results, an increasing appearance of genetic analysis terms such as “qPCR”, “microarray”, “electrophoresis”, and “Taqman” were observed at the point.

Another example may be gleaned from an event in 2013, the approval of Illumina’s sequencer by US-FDA [23] in 2013. An increasing trend shift was observed at phase 3 in the form of increased frequencies of sequencing-related terms (“miRNA”, “rRNA”), devices (“Illumina”, “MiSeq”), and analysis technique/software (“WGS”, “GWAS”, “geNorm”, “NormFinder”). Although the events in which MiSeq of Illumina was launched in 2011 and HiSeq 2500 of Illumina sequencer was launched in 2012, the social influence of FDA approval has seemed more affect the standardization of research in genomics than the launching of device.

Other shifts of note are: 1) In phase 4, the keywords, such as “nano gram”, “genetics”, “genomics”, “methylation”, “MLST”, and “metagenomics”, in Genetics terminology category showed a significantly increased linear trend (p = .039, Table 4).

From content analysis, we identified the drastic increasing trends in the clinical terminology, such as “clinician”, “therapy”, “diagnosis”, “precision”, and “pathogen”, and especially related to the oncology-related terminology, such as “tumor”, “NSCLC”, “AML”, “GBM”, and “tumor DNA”. 2) From phase 1 to phase 3, there was a trend shift in terms related to gene analysis technology and target genes, with an increasing appearance of terms for smaller size genes from the large ones (e.g., from “genomic DNA”, “DNA” and “chromosomes” to “RNA”, “miRNA”, “rRNA”, “exome”, “tRNA”).

Taking the content analysis and statistical analysis results together, we suggest that these genetics terminologies, especially gene analysis technology including biological objects highly to increase in future trends and could be promising standard research topics in clinical genomics. Plus, considering the results of this study, when selecting standard items with a ramification in clinical genetics, we suggest considering the FDA approval that can increase their use in clinics to prioritize genetic technologies.

### Limitations

As the title says, this study was mainly conducted with network analysis and periodic analysis. And we performed the content analysis and statistical analysis to give scientifically supportive results for the main analysis results. The limitations of each analysis are as follow: (1) For the network analysis results, we reviewed only ten keywords in each modularity. For a more precise interpretation of the results, all the keywords should be reviewed in each cluster in future research. (2) A more objective basis for the relation between period analysis and social events should be provided. (3) For content analysis and statistical analysis, it could be more appropriate to use modularity values rather than keywords characteristics of categories. In future research, if we conduct keyword analysis research considering the limitations, we will be able to improve the quality of research.

## Conclusion

Despite the steep decreasing number of keyword frequency in 2020 caused by the downturn of genomics research because of the pandemic status of COVID-19, the overall research field related to the standard of genomics showed a significantly positive trend from 1975 to September 2020 (R2 > 0.585, Table 3; Fig. 2). In the GLM analysis within a phase, Genomics terminology keywords regarding methylation terminology are showing a significantly increasing trend (P = .039) with clinical terminologies of DNA methylation diseases, such as AML and GBM. Also, from the period analysis results, we revealed other influential issues of genetics, such as the completion of the human genome project in 2003, the approval of NGS by the US-FDA in 2013, the outbreak of the COVID-19 pandemic in 2020, and these social events seem to have considerably influenced the standardization research in genomics. Through this comprehensive network analysis study with a period, contents, and statistical analysis, we could provide various types of information such as the relationship between terminologies, the most influential social issues in a standard of genomics field, and trend shifts in genomics terminology fields. Moreover, we statistically estimated and suggested future trends and provided high-demanding items in international standardization for clinical genetics. Therefore, the genomics trend analysis results of this study can be used as a guidance for directing future standards development efforts in clinical genomics.

## Electronic supplementary material

Below is the link to the electronic supplementary material.

Supplementary Material 1

Supplementary Material 2

## Data Availability

All data generated or analyzed during this study are included in this published article and its supplementary information files.
