# Supplementary material for: Network analysis for estimating standardization trends in genomics using MEDLINE
Source: BMC Med Res Methodol. 2022 Oct 7;22:263. doi: 10.1186/s12874-022-01740-4 (PMC9540045; doi:10.1186/s12874-022-01740-4)
Supplement: Supplementary file 2 — Supplementary Material 2 [file 12874_2022_1740_MOESM2_ESM.docx]

**Supplementary Information**

**Network Analysis for Estimating Standardization Trends in Genomics using MEDLINE**

Eun Bit Bae^1^, Se Jin Nam^2^, Sungin Lee, Sun Ju Ahn^1*^

^1^Institute of Quantum Biophysics, Sungkyunkwan University, Seoul, South Korea

^2^Department of Global Convergence, Sungkyunkwan University, Seoul, South Korea

| Phase | Category | B | SE | t | Sig. | 95% Confidence Interval | |
| --- | --- | --- | --- | --- | --- | --- | --- |
|  |  |  |  |  |  | Lower | Upper |
| Phase 0 | Biology | 1.785 | 1.002 | 1.781 | 0.076 | -0.187 | 3.756 |
|  | General | -0.275 | 0.992 | -0.277 | 0.782 | -2.226 | 1.677 |
|  | Genetics | 0.908 | 0.779 | 1.166 | 0.245 | -0.624 | 2.440 |
|  | Medicine | 0.532 | 0.816 | 0.652 | 0.515 | -1.073 | 2.137 |
|  | Proteomics | 0.066 | 0.932 | 0.071 | 0.943 | -1.768 | 1.901 |
| Phase 1 | Biology | 2.067 | 1.436 | 1.439 | 0.151 | -0.758 | 4.892 |
|  | General | 0.114 | 1.421 | 0.080 | 0.936 | -2.682 | 2.911 |
|  | Genetics | 1.329 | 1.116 | 1.191 | 0.234 | -0.866 | 3.524 |
|  | Medicine | 0.997 | 1.169 | 0.853 | 0.394 | -1.302 | 3.297 |
|  | Proteomics | 0.803 | 1.336 | 0.601 | 0.548 | -1.825 | 3.431 |
| Phase 2 | Biology | 3.112 | 9.724 | 0.320 | 0.749 | -16.017 | 22.242 |
|  | General | -1.146 | 9.625 | -0.119 | 0.905 | -20.082 | 17.790 |
|  | Genetics | 6.368 | 7.555 | 0.843 | 0.400 | -8.494 | 21.231 |
|  | Medicine | -1.780 | 7.914 | -0.225 | 0.822 | -17.350 | 13.789 |
|  | Proteomics | 9.319 | 9.046 | 1.030 | 0.304 | -8.477 | 27.116 |
| Phase 3 | Biology | 3.292 | 8.396 | 0.392 | 0.695 | -13.226 | 19.810 |
|  | General | 7.169 | 8.311 | 0.863 | 0.389 | -9.181 | 23.520 |
|  | Genetics | 7.230 | 6.523 | 1.108 | 0.269 | -5.603 | 20.064 |
|  | Medicine | 2.140 | 6.834 | 0.313 | 0.754 | -11.304 | 15.584 |
|  | Proteomics | 4.570 | 7.811 | 0.585 | 0.559 | -10.796 | 19.937 |
| Phase 4 | Biology | 4.220 | 9.441 | 0.447 | 0.655 | -14.353 | 22.793 |
|  | General | 13.600 | 9.345 | 1.455 | 0.147 | -4.785 | 31.984 |
|  | Genetics | 7.283 | 7.335 | 0.993 | 0.321 | -7.147 | 21.713 |
|  | Medicine | 6.367 | 7.684 | 0.829 | 0.408 | -8.750 | 21.484 |
|  | Proteomics | 1.109 | 8.783 | 0.126 | 0.900 | -16.170 | 18.387 |

**Table S1.** The linear regression analysis results for the academic category.

**
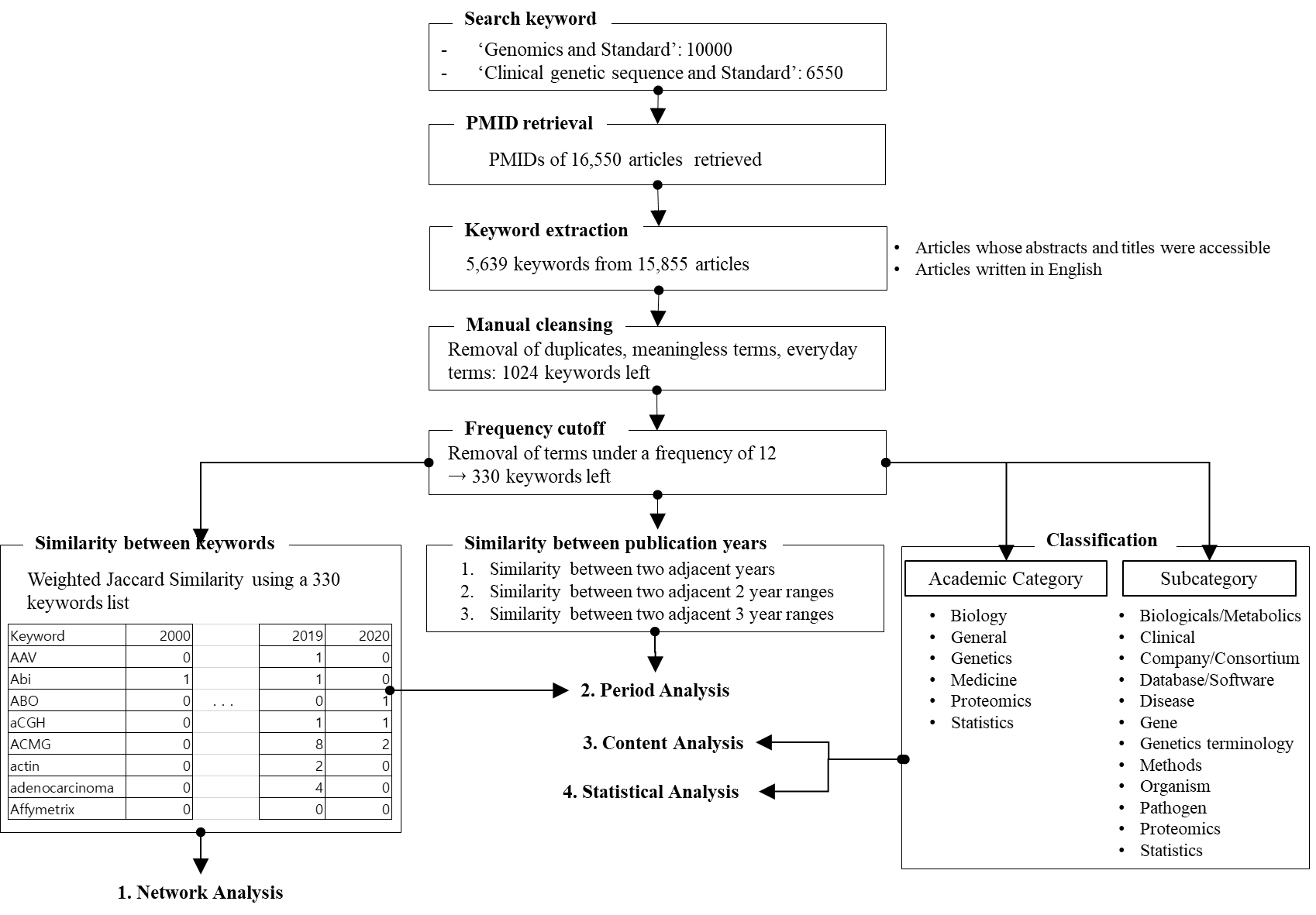
**

**Figure S1.** The study flow from keyword preparation to analysis.


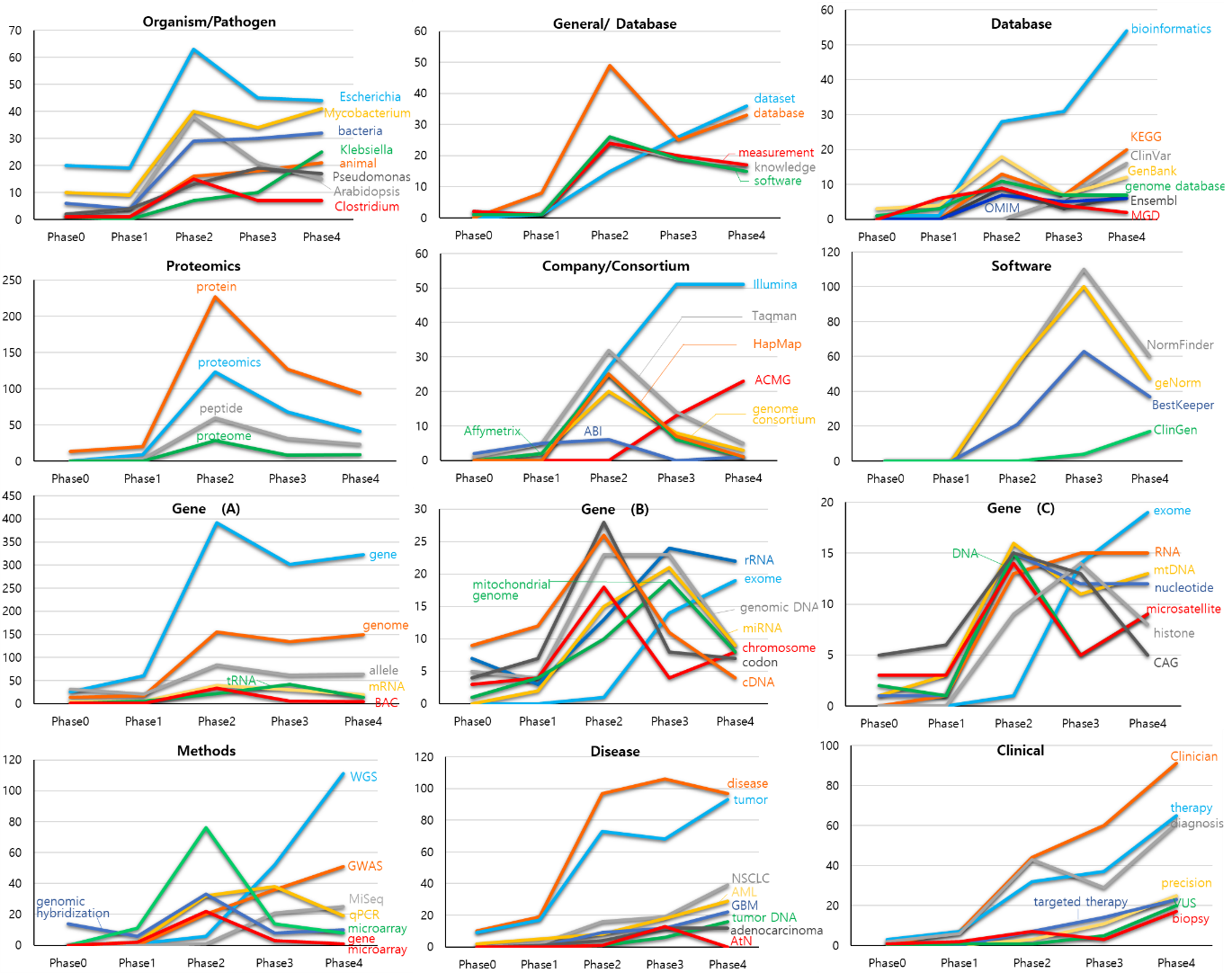


**Figure S2.** The frequency graph of each keyword by category.
